# The weight-adjusted-waist index and cognitive impairment among U.S. older adults: a population-based study

**DOI:** 10.3389/fendo.2023.1276212

**Published:** 2023-11-08

**Authors:** Xiao-tong Huang, Xiang Lv, Hong Jiang

**Affiliations:** Department of Anesthesiology, Shanghai Ninth People’s Hospital, Shanghai Jiao Tong University School of Medicine, Shanghai, China

**Keywords:** a population-based study, weight-adjusted waist index, cognitive function, NHANES, obesity, older

## Abstract

**Objectives:**

Multiple research projects have provided evidence of the correlation between obesity and cognitive impairment. WWI, a novel metric for assessing obesity, has the potential to provide a more precise assessment of muscle and fat mass. This research aimed to investigate the association between WWI and cognitive functioning among elderly individuals residing in the United States.

**Methods:**

This study utilized data from the National Health and Nutrition Examination Survey (NHANES) conducted between 2011 and 2014. Weighted multiple linear regression models, smoothed fitted curves, and generalized weighted models were employed to examine the associations between WWI and cognitive function in linear and nonlinear contexts.

**Results:**

The study included a cohort of 2,764 adult volunteers aged 60 years and older, all with complete data. Upon controlling for all potential confounding variables, our analysis revealed statistically significant negative associations between WWI and the Digit Symbol Substitution Test (DSST) score. Specifically, for each 1-unit increase in WWI, there was a corresponding loss of 3.57 points in the DSST score [-3.57 (-4.31, -2.82)]. The negative correlations between WWI with CERAD total word recall [-0.63 (-0.85, -0.40)], CERAD delayed recall [-0.19 (-0.30, -0.07)], and AFT [-0.65 (-0.94, -0.37)] were significant only in partially adjusted models.

**Conclusion:**

Higher WWI was associated with poorer cognitive function.

## 1 Background

Cognition refers to the cognitive processes through which the human brain receives, analyses, and internally transforms external information, facilitating knowledge acquisition and application. The cognitive abilities encompassed within this framework consist of memory, language, executive, visuospatial, comprehension judgment and computational (1). Cognitive impairment is the deterioration of one or more cognitive functions, resulting in adverse effects on an individual’s everyday functioning and social abilities. Cognitive impairment is prevalent among the elderly population, as evidenced by a study conducted in the United States, which revealed that approximately 33% of individuals aged 65 and above exhibit symptoms of dementia or mild cognitive impairment (MCI) (2).

Obesity, a burgeoning global problem, not only heightens the risk of cardiovascular disease but also exerts a detrimental influence on the central nervous system and cognitive function (3). A substantial body of data indicates a connection between obesity and moderate cognitive impairment, as well as notable changes in the structure and function of the hippocampus. Moreover, a robust correlation has been established between obesity and the specific dementia subtype known as Alzheimer’s disease (4–6). There was a correlation between central adiposity and suboptimal performance on assessments that evaluate executive functioning and visuomotor skills (7). Typically, the evaluation of obesity involves the utilization of body mass index (BMI) and waist circumference (WC). However, it is worth noting that these measurements do not allow for the differentiation between muscle mass and adipose tissue.

The weight-adjusted waist index (WWI) is a novel measure of obesity proposed by Park et al. This index incorporates body composition variations, including muscle and fat mass, providing a more comprehensive assessment of centripetal obesity (8).

There is existing evidence demonstrating a correlation between obesity and cognitive impairment. However, the relationship between WWI and cognitive function remains unexplored in the literature. Hence, this research aimed to investigate the correlation between WWI and cognitive abilities among the elderly population in the United States, utilizing data obtained from the National Health and Nutrition Examination Survey (NHANES).

## 2 Methods

### 2.1 Study population

**Figure 1:**
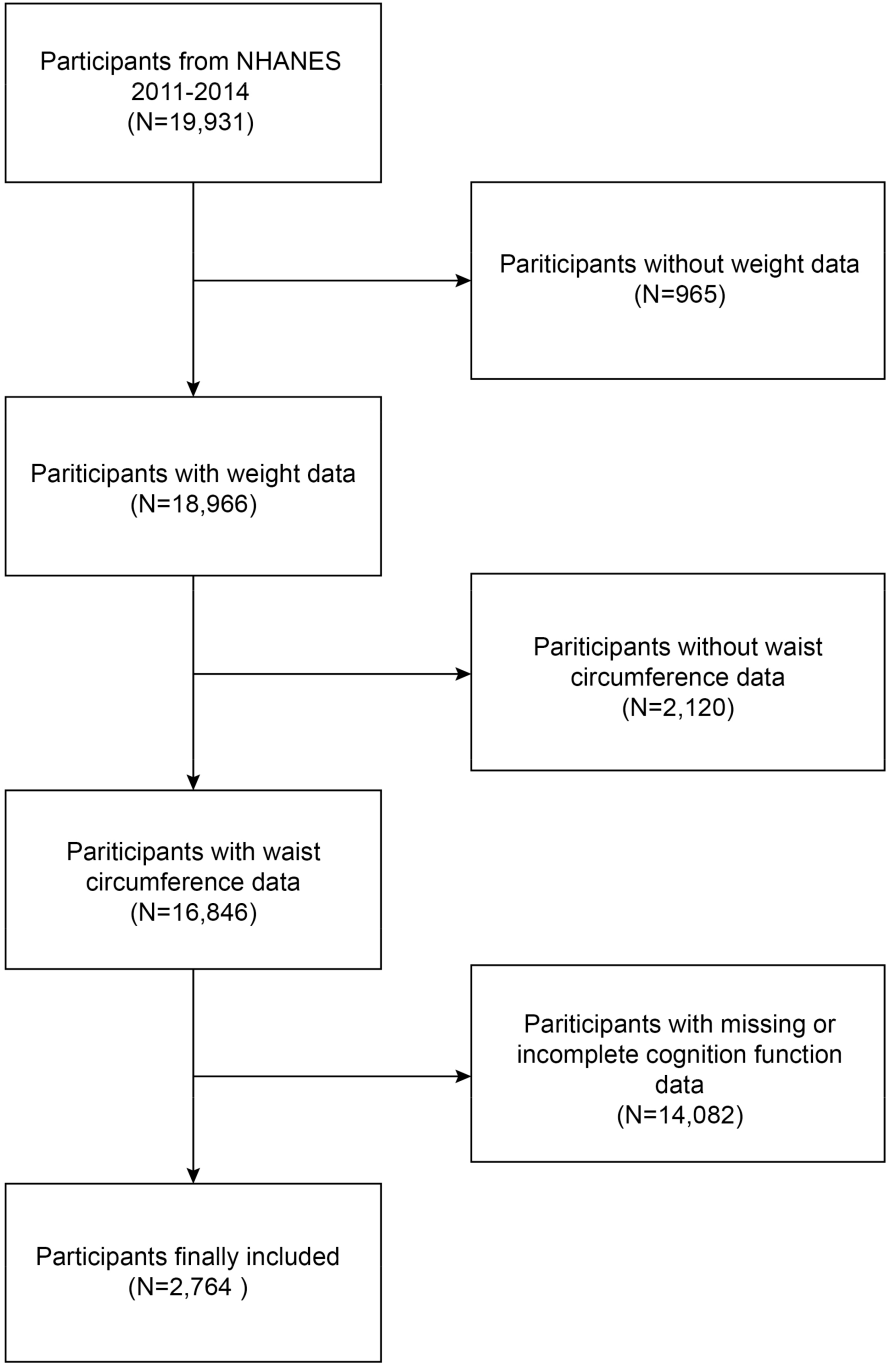
Flow chart of participants selection. NHANES, National Health and Nutrition Examination Survey.

The National Health and Nutrition Examination Study (NHANES) is a continuous and comprehensive countrywide study that seeks to assess the nutritional and health conditions of the population in the United States (9). The Research Ethics Review Board of the National Center for Health Statistics (NCHS) authorized the study procedure. During the recruitment process, all participants were granted written consent. Written consent was obtained from all participants recruited from the NHANES 2011-2014 at the time of their recruitment. Our analysis included participants with thorough information on cognitive functioning assessments and WWI. Initially, a cohort of 19,931 individuals was enrolled. However, after the removal of participants with missing data on weight (n 965), waist circumference (n 2,120), and cognition function (n 1,4082), our final analysis contained a total of 2,764 eligible participants (**Figure 1**).

### 2.2 Weight-adjusted-waist index

With a focus on estimating obesity, the WWI is an anthropometric index based on weight and waist circumference. A more excellent WWI score was indicative of a heightened level of obesity. Health technicians who had received specialized training took data on waist circumference (WC) and weight within the Mobile Examination Center (MEC). The WWI index for each participant was calculated by separating the waist circumference (WC) in centimeters by the square root of the weight in kilograms and afterward rounding the resulting value to two decimal points (8). To facilitate our research, WWI was considered a continuous variable, and individuals were classified based on the tertiles of WWI. WWI was intended to serve as an exposure variable in our research.

### 2.3 Measurement of cognitive function

The Consortium to Establish a Registry for Alzheimer’s Disease (CERAD W-L) evaluates the capacity of individuals to acquire and retain new verbal material in terms of immediate and delayed learning (10, 11). The CERAD W-L protocol comprises three successive learning trials and a delayed recall task. During the three learning trials, participants were provided with instructions to orally articulate a set of 10 words that were not related to each other. Participants tried to recall as many words as they could immediately after the terms were presented. The delayed recall was conducted roughly 10 minutes following the commencement of the word learning trials. Each trial is assigned a maximum score of 10, and the final score for the word list is 40, representing the combined sum of the three tests and the delayed recall.

The Animal Fluency Test assesses verbal category fluency, a fundamental aspect of executive function. Additionally, this test evaluates other abilities related to semantic memory and processing speed (12). With a score awarded for each animal named, participants were instructed to list as many creatures as possible in a minute.

The Digit Symbol Substitution Test (DSST) is a comprehensive assessment tool for evaluating brain health, encompassing various cognitive domains such as visual scanning, processing speed, short-term memory, and sustained attention (13, 14). A paper form is used for the test, and the top of the state has a key with nine numbers and different symbols paired with them. The participants were given a time limit of two minutes to replicate the characters that were associated with the 133 boxes adjacent to the numbers.

### 2.4 Covariables

The covariates considered in the study encompassed age (measured in years), gender (categorized as male or female), race (including Mexican American, Other Hispanic, Non-Hispanic White, Non-Hispanic Black, and Other races), education level (classified as below high school, high school, or above high school), income-to-poverty ratio (PIR), smoking status (indicated as yes or no), diabetes status (shown as yes or no), stroke status (marked as yes or no), blood pressure status(indicated as yes or no), dyslipidemia status(indicated as yes or no), CVD status(indicated as yes or no) and alcohol use status (categorized as more than or less than 12 drinks per year). The NHANES Survey Methods and Analysis Guide provides complete data on various methods employed for collecting variables (https://www.cdc.gov/nchs/nhanes/AnalyticGuidelines).

### 2.5 Statistical analysis

R (version 4.2) and EmpowerStats (version 4.1) were used for all analyses. The sample groups were divided into WWI tertiles, and the demographic traits of each group were assessed using the chi-square test and the t-test. We investigated the linear relationships between WWI and cognitive function using weighted multivariate linear regression models. After logistic transformation, the linear trend of the four dimensions of cognitive function variables was examined using the Cochran-Armitage test. The non-linear relationships between WWI and cognitive function were investigated using additional generalized additive models and smoothed curve fits. To investigate the relationship between WWI and cognitive function among different demographic groups, subgroup analysis and interaction tests were implemented. The threshold for statistical significance was a two-tailed P value of 0.05.

## 3 Results

### 3.1 Baseline characteristics

The population characteristics are shown in **Table 1** in order of WWI tertiles. During the evaluation period, the study included a cohort of 2,764 individuals who were 60 years of age or older. Among this group, 48.95% were identified as male, while 51.05% were identified as female. The average age (SD) and average WWI (SD) of the 2,764 participants were 69.27 (6.74) years and 11.49 (0.71), respectively. WWI tertiles 1–3 ranges were 9.02–11.18, 11.18–11.77, and 11.77–14.79. In contrast to the group exhibiting the lowest WWI, individuals in the group demonstrating the greatest WWI displayed a higher likelihood of being female, belonging to Mexican American or other Hispanic ethnic backgrounds, possessing advanced age, engaging in smoking habits, and exhibiting a greater frequency of diabetes, stroke, and cardiovascular disease, such as hypertension, dyslipidemia, CVD. Additionally, there was a higher probability of individuals having lower levels of educational achievement and household income. Meanwhile, the CERAD-total, CERAD-delayed, Animal Fluency test, DSST scores were lower (**Table 1**).

**Table 1:** Basic characteristics of participants by weight-adjusted-waist index tertiles among U.S. older adults.

Characteristics	Weight-adjusted-waist index			*P*-value
	T1 (9.02-11.18)	T2 (11.18-11.77)	T3 (11.77-14.79)	
Age (years)	67.48 ± 6.32	69.49 ± 6.48	70.30 ± 6.72	<0.0001
Gender, (%)				<0.0001
Male	50.50	50.02	36.54	
Female	49.50	49.98	63.46	
Race/ethnicity, (%)				0.0006
Mexican American	2.00	4.49	4.05	
Other Hispanic	2.31	4.04	5.06	
Non-Hispanic White	80.32	78.33	79.17	
Non-Hispanic Black	10.29	7.94	6.56	
Other races	5.08	5.20	5.17	
Education level, (%)				<0.0001
< high school	11.48	14.91	21.00	
High school	18.76	22.59	24.76	
> high school	69.77	62.5	54.25	
Smoking, (%)				0.0096
Yes	48.04	54.79	49.35	
No	51.96	45.21	50.65	
Stroke, (%)				0.0002
Yes	4.90	4.17	8.36	
No	95.10	95.83	91.64	
Diabetes, (%)				<0.0001
Yes	12.05	22.41	35.42	
No	87.95	77.59	64.58	
HBP, (%)				<0.0001
Yes	45.44	60.44	69.60	
No	54.56	39.56	30.40	
Dyslipidemia, (%)				<0.0001
Yes	48.99	60.27	66.85	
No	51.01	39.73	33.15	
CVD, (%)				<0.0001
Yes	11.87	17.72	21.58	
No	88.13	82.28	78.42	
Alcohol intake ≥12 drinks/year, (%)				<0.0001
Yes	77.30	76.12	66.69	
No	22.70	23.88	33.31	
Family PIR	3.39 ± 1.51	3.16 ± 1.52	2.66 ± 1.51	<0.0001
CERAD total word recall	20.47 ± 4.40	19.70 ± 4.40	19.29 ± 4.41	<0.0001
CERAD delayed recall	6.52 ± 2.18	6.24 ± 2.26	6.11 ± 2.32	0.0003
Animal fluency test	19.16 ± 5.82	18.14 ± 5.40	17.41 ± 5.62	<0.0001
Digit symbol substitution test	55.84 ± 16.13	53.07 ± 16.12	48.82 ± 16.52	<0.0001

Mean ± SD for continuous variables: the P value was calculated by the weighted linear regression model;

(%) for categorical variables: the P value was calculated by the weighted chi-square test.

PIR, the ratio of income to poverty; CERAD, Consortium to Establish a Registry for Alzheimer’s Disease; T, tertiles; HBP, high blood pressure; CVD, cardiovascular disease.

### 3.2 Association between WWI and cognitive function

WWI had a strong negative linear correlation with the Digit symbol substitution test in all three linear regression models. When key demographic factors were adjusted, each unit increase in WWI score was linked to a 3.57-scores decrease in Digit symbol substitution test [-3.57 (-4.31, -2.82)], and a 0.63-scores decrease in CERAD total word recall [-0.63 (-0.85, -0.40)], and a 0.19-scores decrease in CERAD delayed recall [-0.19 (-0.30, -0.07)] and a 0.65-scores decrease in AFT [-0.65 (-0.94, -0.37)], respectively. In the fully adjusted model, the negative correlation of WWI with the Digit symbol substitution test was then 1.34 scores for each increase in WWI score for the Digit symbol substitution test [-1.34 (-2.05, -0.62)]. However, the significant negative linear connection between WWI and CERAD total word recall, CERAD delayed recall, and AFT was not preserved after controlling for all variables (**Table 2**).

**Table 2:** Associations between weight-adjusted-waist index with cognitive function.

Cognitive function	Model 1 [β (95%CI)]	Model 2 [β (95%CI)]	Model 3 [β (95%CI)]
CERAD total word recall	-0.91 (-1.15, -0.68)	-0.63 (-0.85, -0.40)	-0.34 (-0.58, -0.10)
T1	ref	ref	ref
T2	-0.77 (-1.18, -0.37)	-0.28 (-0.66, 0.10)	-0.11 (-0.49, 0.27)
T3	-0.84(-1.43, -0.26)	-0.57 (-1.14, -0.00)	0.03 (-0.54, 0.60)
P for trend	<0.0001	0.0002	0.1979
CERAD delayed recall	-0.33 (-0.45, -0.21)	-0.19 (-0.30, -0.07)	-0.08 (-0.20, 0.05)
T1	ref	ref	ref
T2	-0.28 (-0.49, -0.07)	-0.04 (-0.24, 0.15)	0.04 (-0.15, 0.24)
T3	-0.41 (-0.61, -0.21)	-0.19 (-0.38, 0.01)	0.00 (-0.20, 0.21)
P for trend	<0.001	0.0619	0.9875
Animal fluency test	-1.17 (-1.47, -0.87)	-0.65 (-0.94, -0.37)	-0.15 (-0.44, 0.15)
T1	ref	ref	ref
T2	-1.02 (-1.53, -0.51)	-0.45 (-0.92, 0.03)	-0.12 (-0.59, 0.35)
T3	-1.75 (-2.26, -1.24)	-0.97 (-1.45, -0.49)	-0.20 (-0.69, 0.28)
P for trend	<0.0001	<0.0001	0.4106
Digit symbol substitution test	-5.15 (-6.01, -4.29)	-3.57 (-4.31, -2.82)	-1.34 (-2.05, -0.62)
T1	ref	ref	ref
T2	-2.77(-4.25, -1.28)	-0.34 (-1.59, 0.92)	0.68 (-0.45, 1.81)
T3	-7.01 (-8.48, -5.54)	-4.53 (-5.79, -3.27)	-1.19 (-2.37, -0.01)
P for trend	<0.0001	<0.0001	0.0521

Model 1: no covariates were adjusted. Model 2: age, gender, and race were adjusted. Model 3: age, gender, race, education level, PIR, smoking status, diabetes status, stroke status, HBP status, Dyslipidemia status, CVD status, alcohol using status were adjusted.

PIR, the ratio of income to poverty; CERAD, Consortium to Establish a Registry for Alzheimer’s Disease; T, tertiles; HBP, high blood pressure; CVD, cardiovascular disease.

**Figure 2:**
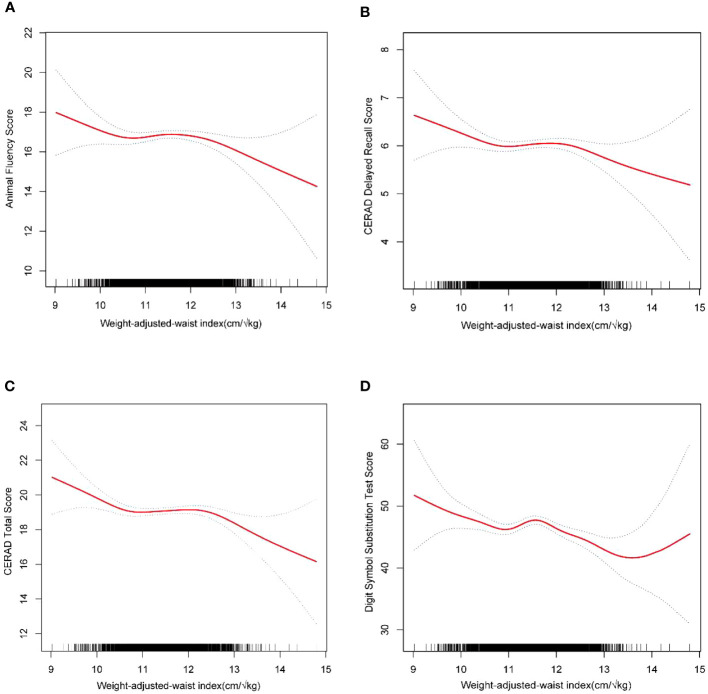
The nonlinear associations between WWI and Digit symbol substitution test. The solid red line represents the smooth curve fit between variables. Blue bands represent the 95% of confidence interval from the fit. **(A)** WWI and Animal fluent test; **(B)** WWI and CERAD delayed recall score; **(C)** WWI and CERAD total score; **(D)** WWI and Digit symbol substitution test.

Additionally, from a nonlinear standpoint, the generalized model and smoothed curve fitting results further corroborated the negative association between WWI and the Digit symbol substitution exam (**Figure 2**).

To assess the uniformity of the association between WWI and cognitive function throughout the general population and to discover potential differences among particular population groups, we carried out subgroup analyses and interaction tests, categorized by age, gender, race, stroke status, diabetes status, HBP status, dyslipidemia status and CVD status (**Table 3**). The findings of this study indicate a notable disparity in the relationship between WWI and cognitive function among individuals who have suffered a stroke. Specifically, participants who have experienced a stroke [-4.37 (-7.42, -1.32)] have a stronger negative correlation between WWI with cognitive function than those without stroke [-1.18 (-1.91, -0.45)]. In the other groupings, the association between WWI and cognitive function persisted (P for interaction > 0.05).

**Table 3:** Subgroup analysis of the association between weight-adjusted-waist index with cognitive function.

Subgroup	CERAD total word recall β (95%CI)	P for interaction	CERAD delayed recall β (95%CI)	P for interaction	Animal fluency test β (95%CI)	P for interaction	Digit symbol substitution test β (95%CI)	P for interaction
Gender		0.6712		0.7351		0.7196		0.0185
Male	-0.44 (-0.82, -0.05)		-0.07 (-0.27, 0.13)		-0.11 (-0.58, 0.36)		-2.47 (-3.60, -1.33)	
Female	-0.33 (-0.64, -0.02)		-0.11 (-0.27, 0.05)		-0.22 (-0.60, 0.16)		-0.72 (-1.64, 0.20)	
Age		0.9020		0.3823		0.3084		0.3553
< 70	-0.39 (-0.71, -0.07)		-0.08 (-0.25, 0.09)		-0.38 (-0.77, 0.01)		-2.10 (-3.07, -1.13)	
≥ 70	-0.42 (-0.78, -0.06)		-0.19 (-0.38, -0.00)		-0.08 (-0.52, 0.37)		-1.42 (-2.50, -0.34)	
Race/ethnicity		0.4695		0.9508		0.3645		0.3814
Mexican American	-0.10 (-1.33, 1.12)		0.02 (-0.61, 0.66)		-0.68 (-2.19, 0.82)		-1.30 (-4.93, 2.34)	
Other Hispanic	0.44 (-1.08, 1.96)		0.16 (-0.63, 0.95)		0.32 (-1.55, 2.19)		2.11 (-2.41, 6.63)	
Non-Hispanic White	-0.39 (-0.66, -0.12)		-0.07 (-0.21, 0.08)		-0.21 (-0.54, 0.13)		-1.54 (-2.35, -0.74)	
Non-Hispanic Black	-0.44 (-1.26, 0.37)		-0.18 (-0.60, 0.24)		0.08 (-0.92, 1.08)		0.08 (-2.34, 2.50)	
Other races	0.53 (-0.64, 1.70)		-0.10 (-0.70, 0.51)		1.16 (-0.28, 2.59)		-2.24 (-5.70, 1.23)	
Stroke		0.0184		0.2800		0.2678		0.0446
Yes	-1.54 (-2.57, -0.52)		-0.37 (-0.90, 0.17)		-0.84 (-2.10, 0.42)		-4.37 (-7.42, -1.32)	
No	-0.28 (-0.53, -0.04)		-0.07 (-0.19, 0.06)		-0.11 (-0.42, 0.19)		-1.18 (-1.91, -0.45)	
Diabetes, (%)		0.6769		0.1337		0.5010		0.2925
Yes	-0.24 (-0.75, 0.27)		0.11 (-0.16, 0.37)		0.07 (-0.56, 0.70)		-0.57 (-2.09, 0.95)	
No	-0.36 (-0.64, -0.09)		-0.12 (-0.26, 0.02)		-0.17 (-0.51, 0.16)		-1.49 (-2.30, -0.68)	
HBP, (%)		0.3329		0.9285		0.1641		0.0162
Yes	-0.24 (-0.55, 0.08)		-0.08 (-0.25, 0.08)		-0.29 (-0.67, 0.10)		-2.14 (-3.07, -1.20)	
No	-0.48 (-0.85, -0.10)		-0.07 (-0.27, 0.12)		0.14 (-0.32, 0.60)		-0.37 (-1.48, 0.75)	
Dyslipidemia, (%)		0.2815		0.3003		0.0793		0.1419
Yes	-0.22 (-0.55, 0.11)		-0.03 (-0.20, 0.14)		0.11 (-0.30, 0.51)		-0.86 (-1.84, 0.12)	
No	-0.48 (-0.83, -0.13)		-0.16 (-0.34, 0.02)		-0.42 (-0.85, 0.01)		-1.93 (-2.97, -0.89)	
CVD, (%)		0.2051		0.0439		0.7011		0.1447
Yes	0.02 (-0.58, 0.62)		0.21 (-0.10, 0.52)		-0.00 (-0.74, 0.74)		-0.14 (-1.93, 1.65)	
No	-0.40 (-0.67, -0.14)		-0.13 (-0.27, 0.00)		-0.16 (-0.48, 0.16)		-1.58 (-2.36, -0.80)	

Age, gender, race, education level, PIR, smoking status, diabetes status, stroke status, HBP status, Dyslipidemia status, CVD status, alcohol using status were adjusted.

CERAD, Consortium to Establish a Registry for Alzheimer’s Disease. HBP, high blood pressure; CVD, cardiovascular disease.

## 4 Discussion

This study aimed to investigate the association between a newly developed indicator of obesity, the Weight-to-Waist Index (WWI), and cognitive function in a sample of 2764 older individuals in the United States. According to our findings, a higher WWI is substantially linked to more severe cognitive impairment. The study found a negative correlation between higher levels of WWI and lower scores on the Digit Symbol Substitution Test (DSST), widely recognized as a reliable measure of overall cognitive function. This association was observed consistently across various subgroups, suggesting that higher levels of WWI are linked to poorer executive function and processing speed. The results of this study indicate that WWI could potentially serve as a reliable measure for evaluating the correlation between obesity and cognitive impairment.

To the extent of our current understanding, this is a pioneering study to evaluate the correlation between WWI and cognitive function, highlighting the negative correlation between higher WWI levels and worsening cognitive function. Obesity has been found to have a detrimental effect on cognitive performance in earlier investigations. Greater BMI was linked to worse cognitive scores, and greater BMI at baseline was also linked to higher levels of cognitive decline at follow-up, according to the prospective cohort research of 2223 middle-aged adults conducted by M Cournot et al. (15). Another cross-sectional study involving 3179 American adults over the age of 60 discovered that those who met the criteria for the metabolic syndrome—abdominal obesity, high triglycerides, and low HDL cholesterol—showed 2.39 fewer points on the CERAD delayed recall test than those who did not [Beta = 2.39, SE = 0.46, 4.32 (0.49) *vs*. 6.71 (0.30)] (16). In our research’s raw and adjusted models, we found a negative linear correlation between WWI and lower DSST scores. The sensitivity analysis using WWI as a tertile also showed a dose-response association between WWI and DSST score. The non-linear relationship perspective further substantiates the negative correlation between WWI and DSST.

Obesity is a state caused by excessive accumulation of body fat. The link between obesity and cognitive dysfunction can be attributed to the metabolic consequences of visceral fat, the metabolic syndrome, including insulin resistance, dyslipidemia, and hypertension (17). The most likely diseases linked to cognitive impairment and neuroinflammation are insulin resistance and hyper-inflammation (18, 19). Adipose tissue releases pro-inflammatory factors (TNF-α, IL-1β, IL-6, MCP1) and inflammation-associated proteins (C-reactive protein, CRP), and these mediators contribute to systemic inflammation (20). Several studies have elaborated on the correlation between systemic inflammation and neuronal degeneration (21, 22). Most of these inflammatory mediators and insulin resistance contribute to mitochondrial malfunction, oxidative stress, and apoptosis, inducing neuronal loss (23, 24). The interplay of inflammatory cells, inflammatory factors, and microglia activation can lead to damage in the hippocampal region and impairment of cognitive learning (25). Gang Ma et al. used liver ischemia-reperfusion as a model to investigate whether ribonuclease could attenuate postoperative cognitive dysfunction and found that surgical trauma considerably enhanced the expression of inflammatory mediators IL-1 and IL-6 in the serum and hippocampus of rats (26). Insulin resistance usually occurs due to increased visceral obesity in excess, and the relationship between insulin resistance and impaired cognitive function may be linked to insulin-degrading enzymes (IDE). These enzymes are involved in the metabolism of both insulin and β-amyloid (27, 28). The deposition of beta-amyloid in the brain is hypothesized to be among the initial, observable indications in the advancement of Alzheimer’s disease, and it is linked to cognitive deterioration, neurodegeneration, and impaired synaptic function (29–31). According to several research, the relationship between obesity and worse cognitive function may involve structural and functional modifications to the cortex and subcortex. Cognitive domains, including working memory, verbal memory, processing speed, and fluid intelligence, demonstrate observable impacts (32–34). In a study conducted by M. E. Levine et al., it was shown that individuals aged 70 years and above who had sarcopenia and obesity, either independently or simultaneously, experienced a decline in cognitive function compared to older adults who did not have sarcopenia or obesity (35).

WWI is an anthropometric index that evaluates elevated body fat mass and indicates reduced muscle mass. The study conducted by Kim et al. revealed a positive correlation between WWI and abdominal fat measures, while a negative correlation was observed between WWI and abdominal muscle mass values (36). In comparison to BMI and WC, WWI provides a more accurate measurement of natural obesity because BMI cannot differentiate between muscle mass and fat mass. A cross-sectional study involving 10,289 Chinese participants with hypertension showed a linear positive association between WWI and dementia (odds ratio [OR], 1.45; 95% CI: 1.35, 1.56), a negative association with MMSE scores (β, -1.09; 95% confidence interval [CI]. -1.24, -0.94). There was a dose-response relationship between WWI and MMSE scores and dementia. In daily clinical practice, WWI can function as a simple and reliable tool for assessing the risk of dementia (37). According to previous research, SIRT1 is substantially downregulated in the hippocampal neurons of mice with cognitive impairment (38). The anti-aging gene Silent information regulation 1 (SIRT1) regulates glycolipid metabolism, inflammatory response, cell death and apoptosis, oxidative stress, and tumor formation. SIRT1 inhibits apoptosis mediated by p53 by dephosphorylating the Lys382 residue of the p53 protein. SIRT1 and p53 may interact to modulate adipocytokines and immune responses, which may be crucial for NAFLD, obesity, and neurodegenerative diseases. Repression of SIRT1 in conjunction with other anti-aging genes, including klotho, p66Shc, and FOXO1/FOXO3a, results in aberrant regulation of glucose, lipid, and amyloid metabolism, which is associated with programmed cell death in the liver and brain. Neurons in a brain with inhibited SIRT1 may undergo premature programmed cell death accompanied by altered astrocyte-neuron interactions, thereby accelerating brain aging (39–42). The WWI may detect and diagnose early-onset anti-aging gene inactivation in the US population, which has implications for the development of obesity, diabetes, and cognitive impairment.

The findings from the subgroup analysis revealed that the association between WWI and cognitive function exhibited variability among different subgroups of stroke patients. Cognitive impairment is a prevalent complication following a stroke, affecting around 60% of those who have survived a stroke within the first year. Moreover, the incidence of cognitive impairment tends to be significantly higher in the immediate aftermath of a stroke (43, 44). 44% of stroke patients admitted to hospitals reported general cognitive impairment, according to data compiled by the Stroke and Cognition Consortium (STROKOG) from 13 studies conducted in 8 different countries (45). Based on longitudinal research, it has been observed that approximately 20% of individuals who have experienced a stroke either initially or previously are susceptible to developing early-onset post-stroke dementia (PSD). Furthermore, the prevalence of late-onset PSD ranges from 4.4% to 23.9% (46).

One of the notable aspects of this study is its comprehensive analysis of the linear and nonlinear correlations between WWI and four distinct cognitive function assessment tools. This approach provides valuable insights into the consistency of the relationship between WWI and cognitive function, offering a multifaceted perspective on the subject matter. Studies have shown that American older adults aged 50 years and older report higher severity of subjective cognitive impairment than Chinese older adults, indicating that the US older individuals may be more sensitive to cognitive impairment when compared with other populations (47). Furthermore, our study incorporated a total of 2,764 individuals who were selected as nationally representative participants based on specific criteria. The ample sample size enabled us to conduct subgroup analyses, thereby facilitating the examination of the relationship between WWI and cognitive function across various populations. This approach enhances the statistical robustness and credibility of our findings. The cross-sectional design of this study limits our ability to demonstrate a causal link between WWI and cognitive performance. The significant adverse effects of obesity on cognitive abilities such processing speed, sustained attention, and working memory, as well as the presence of confounding factors, may be responsible for the observed disparity in the correlation between WWI and the CERAD test, the AFT, and the AFT compared to the DSST. The results in the fully adjusted model may also be inaccurate because the variables linked to WWI and cognitive performance were too vast for us to account for all potential confounding factors.

## 5 Conclusion

Our study suggests that a higher WWI might be linked to poorer cognitive function, potentially predicting cognitive dysfunction in individuals aged 60 years and above in the United States. Further investigation is necessary to substantiate the validity of our findings.

## Data availability statement

The original contributions presented in the study are included in the article/supplementary material. further inquiries can be directed to the corresponding author.

## Ethics statement

The studies involving humans were approved by Research Ethics Review Board of the National Center for Health Statistics (NCHS). The studies were conducted in accordance with the local legislation and institutional requirements. The participants provided their written informed consent to participate in this study.

## Author contributions

X-tH: Conceptualization, Data curation, Formal Analysis, Investigation, Methodology, Project administration, Resources, Software, Supervision, Validation, Visualization, Writing – original draft. XL: Conceptualization, Data curation, Formal Analysis, Investigation, Methodology, Project administration, Software, Writing – original draft. HJ: Conceptualization, Funding acquisition, Investigation, Methodology, Resources, Software, Supervision, Validation, Writing – review & editing.
